# The 5-HT_2C_ receptor agonist, lorcaserin, and the 5-HT_6_ receptor antagonist, SB-742457, promote satiety; a microstructural analysis of feeding behaviour

**DOI:** 10.1007/s00213-015-4112-x

**Published:** 2015-10-28

**Authors:** Suzanne Higgs, Alison J. Cooper, Nicholas M. Barnes

**Affiliations:** School of Psychology, University of Birmingham, Edgbaston, Birmingham, B15 2TT UK; School of Clinical and Experimental Medicine, College of Medical and Dental Sciences, University of Birmingham, Edgbaston, Birmingham, B15 2TT UK

**Keywords:** Lorcaserin, 5-HT_2C_ receptor, 5-HT_6_ receptor, Obesity, Feeding behaviour, Microstructural analysis

## Abstract

**Rationale:**

Whilst the FDA-approved anorectic, lorcaserin and various 5-hydroxytryptamine (5-HT)_6_ receptor antagonists reduce feeding, a direct assessment of their impact upon feeding behaviour is less clear. We therefore examined the action of lorcaserin and the clinical-stage developmental candidate 5-HT_6_ receptor antagonist, SB-742457, upon microstructural analysis of licking behaviour. Such analysis provides a rich source of information about the mechanisms controlling food intake.

**Objectives:**

The objective of the present study was to gain insight into the influence upon feeding behaviour of the 5-HT_2C_ receptor agonist, lorcaserin and the developmental 5-HT_6_ receptor antagonist, SB-742457.

**Methods:**

The impact of lorcaserin and SB-742457 upon licking behaviour of non-deprived rats for a glucose solution was assessed using microstructural analysis.

**Results:**

Lorcaserin (0.1–3.0 mg/kg) displayed a dose-dependent ability to reduce glucose consumption via reduction in the number of bouts of licking. A similar action was evident with SB-742457, but only at the lowest dose tested (3.0 mg/kg).

**Conclusions:**

The behavioural actions of both lorcaserin and SB-742457 demonstrate they directly promote satiety.

## Introduction

Obesity is a major public health problem in many countries and gives rise to various co-morbidities including type 2 diabetes, hypertension and dyslipidemia. These sequelae create a considerable burden to the healthcare system (e.g. Ogden et al. 2014) and at least in the USA are second only to tobacco use as a cause of death in under 70 year olds (Danaei et al. 2009). Against this background, the requirement for safe and efficacious anti-obesity drugs is clear.

There is a considerable history of drugs targeting the 5-hydroxytryptamine (5-HT) serotonin system influencing appetite and feeding in pre-clinical models and patients (e.g. Blundell 1977; Vickers and Dourish 2004). For instance, drugs that increase 5-HT neurotransmission such as fenfluramine (and its d-enantiomer, dexfenfluramine) alone or in combination with phentermine (Fen-Phen; e.g. Weintraub et al. 1984), and subsequently, sibutramine (which also increases synaptic levels of noradrenaline and dopamine; McNeely and Goa 1998; Balcioglu and Wurtman 2000) possess clear therapeutic anorectic efficacy in patients (for review, see Halford et al. 2011); however, their adverse effects on the cardiovascular system (e.g. valvulopathy, pulmonary hypertension; Fitzgerald et al. 2000; Rothman et al. 2000; Launay et al. 2002) eventually led to their removal from the market. Investigation concerning which 5-HT receptors mediate the therapeutic actions of drugs like fenfluramine and sibutramine suggested the 5-HT_2C_ receptor as having at least a major role (e.g. Jackson et al. 1997; Vickers et al. 1999; Dutton and Barnes 2006; Higgs et al. 2011; Berglund et al. 2013). Fortunately, the activation of the 5-HT_2C_ receptor does not appear to be associated with the adverse cardiovascular effects allowing direct 5-HT_2C_ receptor agonists to be evaluated for the anorectic efficacy. However, there is a need to avoid concomitant 5-HT_2A_ or 5-HT_2B_ receptor activation as the former results in neuropsychiatric events (e.g. hallucinations; Nichols 2004) and the latter receptor may be responsible for some of the cardiovascular complications arising from drugs like fenfluramine (Fitzgerald et al. 2000; Rothman et al. 2000; Launay et al. 2002). The pharmacophores of the 5-HT_2A_, 5-HT_2B_ and 5-HT_2C_ receptors are structurally similar (for reviews, see Meltzer and Roth 2013; Michino et al. 2015) and hence it has proved challenging to develop 5-HT_2C_ receptor agonists with presumed sufficient selectivity. One such proposed agent, however, is lorcaserin, which displays around 100- and 19-fold selectivity as an agonist for the human 5-HT_2C_ receptor compared to the human 5-HT_2B_ receptor and human 5-HT_2A_ receptor, respectively (functional selectivity assessed by inositol phosphate accumulation via the appropriate receptor; Smith et al. 2008; Thomsen et al. 2008), although a lower selectivity is apparent (12- and 7-fold, respectively) when the relative affinities are compared (Thomsen et al. 2008; it should be noted that in these studies, lorcaserin was a partial agonist at the human recombinant 5-HT_2A_ receptor with an intrinsic activity of 0.75). This discrepancy between the selectivity for the three 5-HT_2_ receptors when comparing agonist potency or receptor affinity may indicate lorcaserin displays biased agonism and/or there were differential levels of receptor reserve apparent when using recombinant expression of the different 5-HT_2_ receptors. Nevertheless, lorcaserin is now approved by the FDA as a prescription medication to assist weight loss regimens (trade name, Belviq®; http://www.fda.gov/NewsEvents/Newsroom/PressAnnouncements/ucm309993.htm), although a caveat to the approval is the need to perform post-marketing studies to, for example, better examine the risk of major adverse cardiac events. However, despite this recent approval, there are a relatively small number of papers in the literature reporting the actions of lorcaserin in pre-clinical models of obesity and feeding (Smith et al. 2008; Thomsen et al. 2008; Fletcher et al. 2009; Higgins et al. 2014; Burke et al. 2014) and these have only reported lorcaserin’s effect on the quantity of food intake (acute lorcaserin administration) or on body weight (chronic lorcaserin administration). Given the complexity of 5-HT’s action upon feeding, in an attempt to gain further insight into the action of lorcaserin upon feeding behaviour, in the present study, we report the microstructural analysis of lorcaserin’s action upon licking behaviour of rats to ingest a liquid diet. Analysis of licking responses provides a rich source of information concerning the principal factors (including satiety), which determine food intake (e.g. Davis and Smith 1992; Davis et al. 2001). To establish the behavioural mechanisms that underlie drug-induced changes in food intake, the effects of pharmacological manipulations can be compared with available reference standards that display distinctive effects on licking patterns associated with satiety or orosensory variables (e.g. Davis and Smith 1992; Davis et al. 2001). We have previously used this approach successfully to reveal the effects of diverse pharmacological agents on eating with high resolution. For example, we have found that whilst both the cannabinoid CB1 receptor antagonist, rimonabant and the 5-HT mimetic, sibutramine, both decreased total intake of glucose, this was achieved by different mechanisms; rimonabant primarily decreased licking bout duration (Higgs et al. 2003) whereas sibutramine decreased bout number (Higgs et al. 2011). Such data indicates that cannabinoid neurotransmission is important for food palatability whereas 5-HT neurotransmission influences satiety. Identification of such mechanistic differentiation highlights the usefulness of microstructural analysis of feeding behaviour to better understand the mechanisms of drug-induced changes in food intake.

This present report is extended further to also assess in the behavioural action of a selective 5-HT_6_ receptor antagonist, SB-742457, using the microstructural analysis of licking behaviour. The 5-HT_6_ receptor has also been proposed as a suitable therapeutic target to reduce obesity although the peer-reviewed reports simply assess the ability of 5-HT_6_ receptor ligands to modify the quantity of food intake or body weight (Woolley et al. 2001; Fisas et al. 2006; Garfield et al. 2014).

## Materials and methods

### Animals

For each compound tested, 12 drug naïve adult male hooded Lister rats (Charles River, UK), weighing approximately 200 g at the beginning of training, were housed in groups of four. Rats were maintained on a 12:12 h light/dark cycle (lights on at 0700 hours), at a constant temperature of 21 ± 1 °C (humidity 50 %). Food pellets (rat and mouse expanded ground maintenance diet; Special Diet services, Cambridge, UK) and water were available ad libitum, except when the animals were in the test chambers. All testing was conducted in the light phase between 0900 and 1700 hours. The experiments were carried out in accordance with the terms of the UK Animals (Experimental Procedures) Act, 1986.

### Drugs

The selective 5-HT_2C_ receptor agonist, lorcaserin (custom synthesis performed by Axon Medchem) was prepared for injection by dissolving in 0.9 % saline and was administered via the i.p. route. The selective 5-HT_6_ receptor antagonist, SB-742457 (Axon Medchem), was homogenised in 1 % methylcellulose and administered orally. Both drugs were administered in a volume of 1.0 ml/kg.

### Apparatus

Testing was carried out using an MS80 multistation lick analysis system (Dilog Instruments, Tallahassee, FL, USA), which has been described in detail previously (Higgs and Cooper 1997). Briefly, rats were placed in a Perspex chamber that had an opening in the centre of the front wall allowing access to a drinking spout that was connected to a plastic 50-ml measuring tube containing the glucose solution (200 mM glucose dissolved in distilled water 200 mM), which is palatable to rats. The lickometer was connected to an amplifier that passed less than 60 nA through the rat every time tongue contact was made with the spout. The current was fed to a standard personal computer (Opus Technology, Surrey, UK), which stored the time of each tongue contact to the nearest millisecond.

### Training

A non-deprivation, free-feeding paradigm was used to avoid the possibility that deprivation might modify licking responses that would complicate identification of independent drug effects. During the training phase, each rat was placed in the test chamber daily with access to the drinking spout to obtain the glucose solution. The rats were left in the test apparatus for 20 min from the time of the first lick at the drinking spout for the glucose solution. This training was repeated daily until each rat achieved a consistent number of total licks (typically achieved by day 5). Although 12 rats in each study (lorcaserin or SB-742457) began training, 1 and 2 rats failed to achieve baseline levels of licking in the lorcaserin and SB-742457 studies, respectively; these rats were not studied further.

To familiarise the rats with the drug administration procedure, on the 2 days before drug testing began, each rat received saline administration via the appropriate route of administration.

### Testing

Administration of vehicle or drug was made using a repeated-measures design, with the order of treatment randomised. After injection, each rat was returned to the home cage before being placed individually in a test chamber, where it had access to the glucose solution for 20 mins. The vehicle/drug administration test interval for the lorcaserin study was 30 and 60 min for the SB-742457 study. ‘Wash-out’ between successive administration of drug/vehicle was at least 48 h.

### Analysis

The accumulated lick data were grouped into bouts defined as the licking occurring with an upper inter-lick interval of less than 400 ms, which corresponds to an interval that is just above twice the average inter-lick interval and is consistent with our previous studies (Higgs and Cooper 1996, 1997, 1998, 2000). Drug impact was examined on four microstructural variables: the number of bouts, the duration of a bout, the intra-bout lick rate (defined as the average number of licks per second within a bout) and the latency to begin licking (timed from the shutter opening allowing access to the drinking spout to the first lick). Analysis of data was by one-way analysis of variance (ANOVA) for repeated measures. The effects of drug relative to vehicle were assessed using a post hoc *t* test corrected for multiple comparisons. For the number of bouts of licking, values were obtained for each rat, and then mean values and standard error of the mean (SEM) were calculated for each treatment. For bout duration and intra-bout lick rate, values across the test period for each rat were averaged, and the mean and SEM were subsequently calculated for treatment groups. Three of the 11 rats in the lorcaserin study failed to register a lick at the highest dose of lorcaserin tested (3.0 mg/kg) and so could not contribute to the microstructural analysis. The data were analysed both without the data from these rats and using multiple imputation to substitute for the missing values. The pattern of results was similar in both cases. For brevity, we just report the results with the affected data points excluded. The latency data were not normally distributed and so the data were square root transformed before analysis. Statistical tests were performed using Sigma Stat (Jandel Corporation, San Rafael, CA, USA, 1992–1994). A result was considered statistically significant if *p* < 0.05.

## Results

### Dose-dependent effect of lorcaserin on licking for glucose solution

**Fig. 1:**
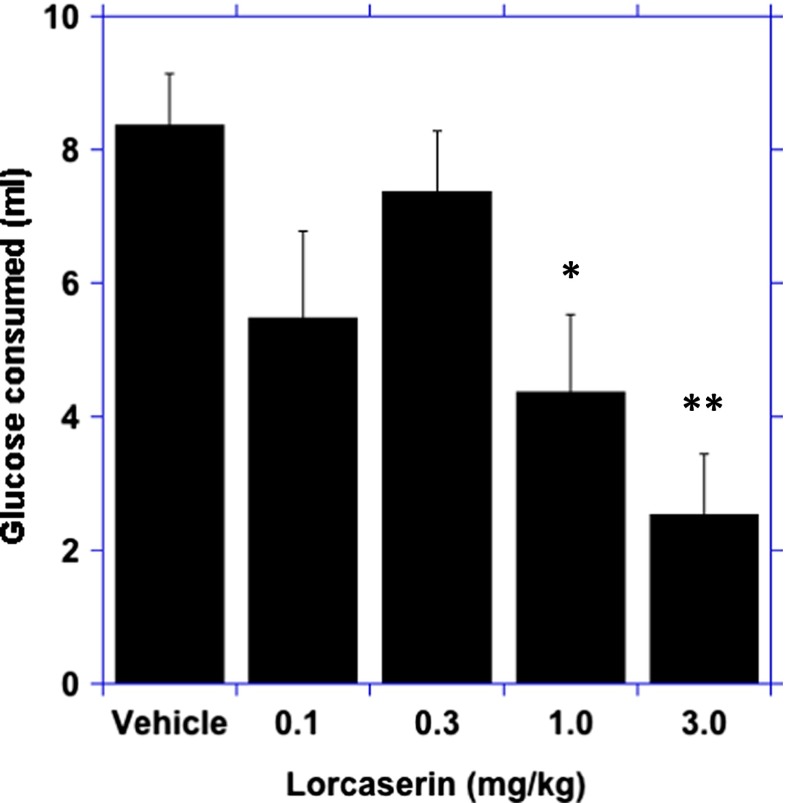
Dose-dependent ability of lorcaserin to reduce the consumption of glucose. Data represent the mean ± SEM, *n* = 12, *p < 0.05, **p < 0.01

There was a main effect of lorcaserin on the amount of glucose solution consumed ((4 ,28) = 6, *p* < 0.001). In comparison to the vehicle response, there was a significant reduction in glucose solution consumed after the 1.0 mg/kg (*p* < 0.05) and 3.0 mg/kg doses of lorcaserin (*p* < 0.01; Fig. 1).

### Microstructural analysis

There was a main effect of lorcaserin on total licks for the glucose solution (*F* (4, 28) = 6; *p* < 0.01; Table 1). In comparison to the vehicle response, there was a significant reduction in the total licks for the glucose solution after the 1.0 and 3.0 mg/kg doses of lorcaserin (*p* < 0.05; Table 1); the lorcaserin-induced reduction in total licks was due to a reduction in the number of lick bouts (*F*(4, 28) = 5.7; *p* < 0.001; Table 1) rather than an effect on lick bout duration (*F*(4, 28) = 1.9; *p* = 0.1; Table 1). There was a main effect of lorcaserin on intra-bout lick rate (*F*(4, 28) = 6; *p* < 0.01) with a significant reduction in intra-bout lick rate but only at the highest dose tested of lorcaserin (3.0 mg/kg; *p* < 0.01; Table 1). There was also a significant effect of lorcaserin on the latency to lick (*F*(4,28) = 4.6, *p* < 0.01); post hoc analysis indicated that latency was significantly increased at the highest does tested (3.0 mg/kg; Table 1).

**Table 1:** Total number of licks and microstructural data after lorcaserin. The microstructural data are shown as means. Standard error difference between means is shown in brackets

Microstructure analysis	Vehicle	Lorcaserin			
		0.1 mg/kg	0.3 mg/kg	1.0 mg/kg	3.0 mg/kg
Number of licks	2091.4 (192.4)	1366.3 (325.7)	1837.3 (231.0)	1087.4* (292.7)	635.3**(224.5)
Bout duration (s)	3.4 (0.4)	3.5 (0.5)	3.3 (0.4)	2.4 (0.4)	2.4 (0.4)
Bout number	98.0 (10.1)	60.5* (13.8)	89.5 (12.1)	59.5 (15.5)*	36.0**(11.4)
Intra-bout lick rate (licks/s)	6.3 (0.2)	6.2 (0. 2)	6.3 (0. 2)	6.6 (0.2)	5.8* (0.2)
Latency (s)	31.5 (17.9)	26.8 (11.2)	25.0 (8.6)	39.7 (18.2)	262.5** (130.1)

Asterisks indicate significantly different from vehicle control condition (*p < 0.05; **p < 0.01)

### Effect of SB-742457 on licking for glucose solution

**Fig. 2:**
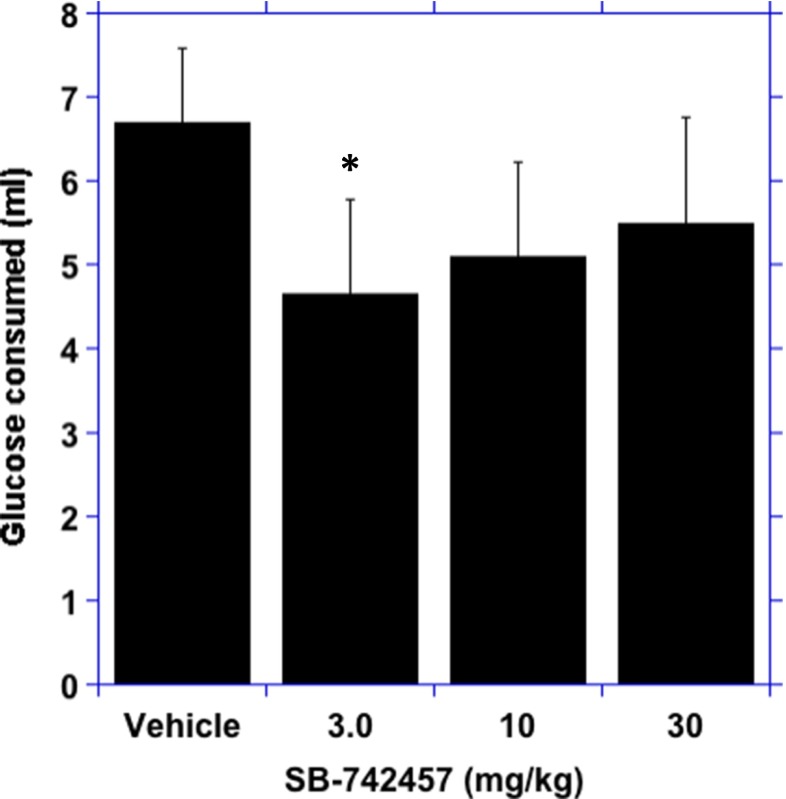
Ability of SB-742457 to reduce consumption of glucose. Data represent the mean ± SEM, *n* = 12, *p < 0.05

There was a main effect of SB-742457 on the amount of glucose solution consumed (*F*(3, 27) = 3.4; *p* < 0.05). In comparison to the vehicle response, there was a significant reduction in glucose solution consumed after the 3.0 mg/kg dose of SB-742457 (*p* < 0.05; Fig. 2).

### Microstructural analysis

There was a significant reduction in total licks for glucose solution after SB-742457 (*F*(3, 27) = 3.3; *p* < 0.05) but this was only significant at the lowest dose tested (3.0 mg/kg; *p* < 0.01; Table 2). The reduction in total licks was due to a decrease in the number of lick bouts (*F*(3, 27) = 2.8; *p* < 0.05; Table 2) rather than bout duration (*F*(3, 27) = 1.8; *p* = 0.2; Table 2). There was a significant main effect of SB-742457 on intra-bout lick rate (*F*(3, 27) = 4.8; *p* < 0.01) but the only significant reduction was at the highest dose tested (30 mg/kg; *p* < 0.01; Table 2). There was no significant effect of SB-742457 on latency to start licking (*F*(3, 27) = 0.14, *p* = 0.9; Table 2).

**Table 2:** Total number of licks and microstructural data after SB-742457. The microstructural data are shown as means. Standard error difference between means is shown in brackets

Microstructure analysis	Vehicle	SB-742457		
		3.0 mg/kg	10 mg/kg	30 mg/kg
Number of licks	1456.7 (199.9)	992.8* (255.2)	1130.7 (249.9)	1238.9 (278.4)
Bout duration (s)	2.7 (0.5)	1.9 (0.1)	2.1 (0.3)	2.0 (0.3)
Bout number	97.2 (12.8)	73.5* (17.2)	80.1 (13.9)	114.5 (27.3)
Intra-bout lick rate (licks/s)	6.4 (0.1)	6.4 (0.2)	6.3 (0.2)	5.9* (0.1)
Latency (s)	84.7 (46.9)	66.1 (28.0)	76.1 (20.7)	62.7 (23.9)

Asterisks indicate significantly different from vehicle control condition (*p < 0.05)

## Discussion

Previous studies exploring the anti-obesity actions of the 5-HT_2C_ receptor agonist, lorcaserin (Smith et al. 2008; Thomsen et al. 2008; Higgins et al. 2014; Burke et al. 2014) or 5-HT_6_ receptor antagonists (Woolley et al. 2001; Garfield et al. 2014) in animal models have utilised food intake or body weight to measure their impact. The present study utilised microstructural analysis of licking behaviour in rats for a liquid diet to offer direct insight into the behavioural mechanisms influenced by lorcaserin and the selective 5-HT_6_ receptor antagonist, SB-742457. By examining the pattern of licking behaviour predictions can be made concerning the influence of drug upon satiety and the palatability of food (Davis and Smith 1992; Breslin et al. 1996; Higgs and Cooper 1996, 1997, 1998; Davis et al. 2001; Cooper and Higgs 2005; Higgs et al. 2011).

In the present study, lorcaserin treatment resulted in a selective reduction in the number of bouts of licking behaviour that was responsible for the reduced food intake. Importantly, the effect on bout number was also observed at doses that had no non-specific effects on licking latency or lick rate. Rats lick at a constant rate of about 6–7 licks/s (Stellar and Hill 1952) and drug-induced disruption of this rate would suggest that a pharmacological treatment might be affecting sensorimotor coordination, as distinct from a specific effect on the controls of ingestion. For example, Davis and Smith (1992) showed that intra-bout lick rate is affected by moving the drinking spout progressively further away from an animal, but not by altering the solution concentration. Hence, the current pattern of results suggests that since lorcaserin was able to decrease bout number in the absence of a reduced intra-lick rate, a modulation of motoric activity did not complicate interpretation. However, it should be noted that the effect was not linear in that the 0.3 mg/kg dose has less of an effect on bout number than the 0.1 mg/kg dose. Similar effects on licking microstructure are observed when delaying negative feedback post-ingestive signals, by adding the non-digestible sugar mannitol to glucose solutions (Davis and Levine 1977). Conversely, the addition of the non-nutritive sweetener saccharin to glucose, which selectively increases palatability, increases bout duration whilst having minimal effects on bout number (Breslin et al. 1996). Hence, selective action of lorcaserin on bout number supports promotion of satiety by the drug rather than an effect on taste palatability, which is consistent with the mechanism of action of some less-selective 5-HT_2C_ receptor agonists in rodents (e.g. Ro 60–0175; Clifton et al. 2000) and humans (Thomas et al. 2014) as well as the 5-HT releasing agents, fenfluramine (d-fenfluramine) and sibutramine (Tallett et al. 2009; Higgs et al. 2011; Burke et al. 2014).

One study that has examined the behavioural effects of lorcaserin assessed its effect on the behavioural satiety sequence (Higgins et al. 2014). It was reported that lorcaserin (0.3–1.0 mg/kg) advanced the behavioural satiety sequence in non-deprived rats consuming a sweetened mash diet over 1 h, consistent with the effect on satiety reported here. Lorcaserin was also reported to reduce deprivation-induced food intake, operant responding for food- and nicotine-motivated behaviours (Higgins et al. 2012). These data suggest that lorcaserin has effects to reduce responding for both food and drug rewards. One explanation for the overall pattern of behavioural responding after lorcaserin is that the drug has a dual mechanism of action to modulate neuronal systems involved in both (1) the processing of metabolic signals related to nutritional state and (2) reward-related processing (Asin et al. 1992; Higgins et al. 2012). Indeed, it may be that both these mechanisms are critical for the observed effects of lorcaserin on food intake. The pleasantness of food is known to decline as food is eaten, a phenomenon known as alliesthesia (Cabanac 1971). It is possible that lorcaserin enhances alliesthesia, such that food becomes less rewarding at a faster rate during a meal, thereby enhancing satiety. This suggestion requires further testing, but such a unifying explanation of the anorectic effects of lorcaserin is consistent with other evidence that points towards important interactions between homeostatic and reward processes in appetite control (Berthoud 2011).

In terms of the underlying neuronal mechanisms for lorcaserin and SB-742457 to promote satiety, elegant neurobiological studies have demonstrated a common population of pro-opiomelanocortin (POMC) neurones within the arcuate nucleus of the hypothalamus that are activated by 5-HT_2C_ receptor agonists (Berglund et al. 2013; Burke et al. 2014) and also d-fenfluramine and sibutramine to signal their anorectic actions (Heisler et al. 2002, 2006; Lam et al. 2008; Xu et al. 2008, 2010; Berglund et al. 2013; Burke et al. 2014); and is consistent with their effects upon satiety (Hillebrand et al. 2002). Results from the Heisler laboratory have also implicated the paraventricular nucleus (PVN) within the hypothalamus as a target for a hypophagic dose of the selective 5-HT_6_ receptor antagonist, SB-399855 (Garfield et al. 2014). Hence, the arcuate-PVN circuit would appear to be important to mediate the hypophagic actions of both 5-HT_2C_ receptor agonists and 5-HT_6_ receptor antagonists; the latter potentially increasing neuronal activity via disinhibition (Garfield et al. 2014, 2015). 5-HT_2C_ receptors are also located on GABA neurons in the VTA and activation of these receptors has been reported to decrease DA release in the nucleus accumbens and frontal cortex (Di Matteo et al. 2002). This latter mechanism may underlie the effects of lorcaserin on reward-related responding.

Lorcaserin’s positive results from three phase III trials (Smith et al. 2010; Fidler et al. 2011; O’Neil et al. 2012; for meta-analysis of the data, see Chan et al. 2013) in the relative absence of adverse side effects, importantly including only a very small additional risk of cardiovascular events (Weissman et al. 2013), led to the FDA approving lorcaserin when combined with a reduced calorie diet and exercise regimen for obese (BMI ≥30) or overweight adults (BMI ≥27) who also have at least one co-morbid condition (e.g. hypertension, type-2 diabetes, dyslipidemia). Despite approval as a medication, there is still much to understand about lorcaserin’s effects on eating behaviour processes; in particular, its effects on reward processes and the relationship of such actions upon satiety. Knowledge of the specific behavioural mechanisms will be helpful in guiding advice and support for patients prescribed the drug, for example in educating them about what to expect from treatment.

Lorcaserin’s impact upon feeding behaviour of rats identified in the present study is consistent with the self-reporting of decreased appetite and hunger of patients receiving the drug (Martin et al. 2011), further supporting the use of microstructural analysis of licking behaviour in pre-clinical models to predict clinical outcome.

Whilst an early report indicated that activation of the 5-HT_6_ receptor may offer therapeutic potential to promote weight loss (Fisas et al. 2006), evidence from the use of various 5-HT_6_ receptor antagonists (Woolley et al. 2001; Garfield et al. 2014; for review, see Heal et al. 2008 that also includes a review of data presented at conferences) and knock-down of 5-HT_6_ receptor gene expression (Woolley et al. 2001) indicates inhibition of 5-HT_6_ receptor signalling possesses the clearest anorectic potential from targeting this receptor. This effect arising from a blockade of 5-HT’s actions contrasts the dogma of 5-HT’s influence upon feeding. Similar to the situation with lorcaserin, data arising from the use of 5-HT_6_ receptor antagonists in feeding studies use quantity of food intake (acute drug administration studies) or body weight (chronic drug administration studies) as the response readouts (Woolley et al. 2001; Garfield et al. 2014; for review, see Heal et al. 2008), although data presented at a conference indicates that the 5-HT_6_ receptor antagonist, PRX-07034 promotes satiety (Gannon et al. 2006; reviewed in Heal et al. 2008). In the present study, we assessed the action of the selective 5-HT_6_ receptor antagonist, SB-742457, using the microstructural analysis of licking behaviour paradigm. SB-742457 was selected for study as it is already a clinical candidate (for Alzheimer’s disease; e.g. Maher-Edwards et al. 2011) and hence would allow relatively rapid translation of the present pre-clinical study into clinical trial. SB-742457 at a dose likely to antagonise central 5-HT_6_ receptors (Idris et al. 2010; Witten et al. 2012) decreased the number of bouts of licking behaviour that was responsible for the reduction in food intake, demonstrating a drug-induced increase in satiety. This effect, however, was only evident at the lowest dose tested, indicating the presence of an inverted bell-shaped response curve for this drug, which has been reported when investigating the action of this drug on other types of behaviour (de Bruin et al. 2013). Similar to the data obtained with lorcaserin, an effect of SB-742457 upon the number of bouts of licking was apparent without an effect upon licking latency or lick rate, excluding motoric effects from influencing the response to drug.

In summary, in the present study the microstructural analysis of ingestive behaviour identified that both the relatively selective 5-HT_2C_ receptor agonist, lorcaserin and the 5-HT_6_ receptor antagonist, SB-742457, displayed an anorectic action by primarily reducing the number of bouts of licking behaviour, which indicates a direct promotion of satiety by both drugs. A better understanding of the behavioural mechanism of action of anti-obesity drugs allows informed decisions to select drug combinations in the search for more efficacious anti-obesity therapies. In addition, for the lorcaserin study, the reverse translation of supporting data from the clinic further validates the predictive value of this behavioural model.
